# Natural climate-change-related crises: a systematic review of organizational and community preparedness and resilience

**DOI:** 10.1186/s12889-026-27846-8

**Published:** 2026-06-18

**Authors:** Anett Wolgast, Donatella Di Marco, Santiago Renedo, Alicia Arenas, Bruna Gomes, Maria Giannacourou, Giedre Kaciene, Tomas Lengemann, Carlos Carvalho, Danguole Rutkauskiene

**Affiliations:** 1 Department of Psychology, University of Applied Sciences FHM Campus Hanover, Lister Straße 17, Hannover, 30163 Germany; 2 Universidad de Sevilla, Seville, Spain; 3 Sparkling Institution, Porto, Portugal; 4 Creative Thinking Development, Attiki, Greece; 5 Vytautas Magnus University, Baltic Education Technology Institute, Vilnius, Lithuania; 6 University of Applied Sciences FHM Bielefeld, Bielefeld, Germany; 7 Baltic Education Technology Institute, Vilnius, Lithuania

**Keywords:** climate change, crisis matrix, natural desasaster, systematic review

## Abstract

**Background:**

Climate change has intensified the frequency and severity of natural disasters, presenting profound challenges to the resilience of communities and organizations. While preparedness and resilience interventions aim to mitigate the impact of these crises by fostering adaptive capacities, the local conditions, diversity, and educational dimensions of such interventions remain underexplored in the literature. This preregistered qualitative systematic review aimed to (a) identify and categorize organizational and community-based preparedness and resilience interventions addressing natural climate change-related crises; (b) map the reported effectiveness of these interventions in enhancing preparedness and resilience; and (c) explore the role of empowering education in strengthening resilience at both the community and organizational levels.

**Methods:**

Following the PRISMA 2020 guidelines, we systematically searched, screened, and mapped quantitative and qualitative primary studies on preparedness and resilience interventions. We included studies addressing natural hazards in community or organizational settings. A total of 2,356 records were screened, and 57 studies met the inclusion criteria.

**Results:**

The thematic synthesis revealed that described interventions often rely on local conditions, community participation, and place-based education. Empowering education increased preparedness, particularly among youth. However, many interventions are short-term, reactive, and lack structural support or codesign.

**Conclusion:**

The reviewed studies support inclusive, context‑sensitive, and sustained approaches to resilience. Across this literature, resilience of communities and organizations facing climate‑related natural hazards is portrayed as emerging from local conditions, social participation, and learning processes rather than from stand‑alone interventions.

**Supplementary Information:**

The online version contains supplementary material available at 10.1186/s12889-026-27846-8.

## Introduction

The accelerating pace of climate change has led to a marked increase in the frequency and severity of natural disasters, presenting profound challenges for both communities and organizations [1]. These crises, ranging from floods and wildfires to hurricanes and droughts, demand robust and adaptive responses that go beyond immediate relief. In this context, preparedness and resilience interventions have emerged as critical strategies aiming at mitigating the adverse impacts of such events [2]. An intervention in this context refers to a structured set of activities, strategies, or measures implemented at the community or organizational level to enhance preparedness and strengthen resilience to climate‑related natural hazards [2]. These interventions seek to strengthen adaptive capacities at multiple levels, enabling systems to anticipate, absorb, and recover from shocks more effectively. While a growing body of research has examined various preparedness and resilience initiatives (e.g., [28, 41, 67*]), a systematic mapping synthesis of qualitative and quantitative evidence remains necessary. There is a limited overview of the specific types of shocks through natural disasters and resilience-relevant responses, as well as (prevention) interventions implemented, their reported effectiveness, and the role that empowering education plays in enhancing resilience. Reported effectiveness in this context refers to the outcomes described in the included studies regarding changes in preparedness, resilience, or related capacities following an intervention, as assessed by the authors using qualitative or quantitative evidence. Empowering education, defined as participatory, inclusive, and capacity-building learning embedded in general education, vocational training, higher education, or lifelong adult education, may serve as a key mechanism for fostering long-term resilience within both organizational and community settings [26].

This qualitative systematic review aimed to address these gaps by pursuing three core objectives. First, we aimed to identify and categorize the types of organizational and community-based reactions to natural disasters, preparedness, and resilience interventions designed to address natural disasters linked to climate change. Second, we aimed to map the reported effectiveness of these interventions in enhancing preparedness and resilience outcomes. Third, this review explored the role of empowering education in strengthening the resilience of communities and organizations, with a particular focus on how educational approaches contribute to adaptive capacity and crisis readiness.

## Theoretical foundation

A crisis in terms of a system shock may be broadly understood as a significant disruption that threatens the stability and functioning of individuals, groups, or systems. Early conceptualizations highlighted the urgency and collective nature of crises, describing them as situations in which all members of a group are confronted with a shared threat [36]. Subsequent perspectives expanded on this by emphasizing the profound psychological and systemic impact of crises, portraying them as disruptions that affect an entire system and challenge its fundamental assumptions and identity [58, 68]. More contemporary definitions have outlined organizational contexts, such as public institutions, schools, or NGOs, where crises jeopardize operational continuity, leadership structures, and institutional viability. These events are typically characterized by ambiguity regarding their causes and consequences, and they often necessitate swift decision-making under pressure [59]. Thus, crises are complex phenomena characterized by a set of defining characteristics that distinguish them from routine disruptions. They are typically urgent, requiring immediate and often high-stakes decision-making.

Not all incidents qualify as crises; only those marked by urgency, uncertainty, and systemic threat meet this threshold [38, 59]. Crisis typologies help guide analysis and response, distinguishing between technological, natural, nuclear, and large-scale social crises [36, 58]. Some crises, such as nuclear accidents or major earthquakes, are partially anticipated but remain difficult to manage due to their systemic nature. Others, such as 9/11 or the COVID-19 pandemic, defy conventional understanding [36].

Furthermore, crises can be described by origin (i.e., internal vs. external and cause, technological-economic vs. social-organizational, [27]). This informs tailored responses, such as the EU Civil Protection Mechanisms, which coordinated 58 activations in 2024. In the public sector, all regions and organizations are exposed to crises [72]. Effective management requires balancing proactive and reactive strategies, with success depending on preparedness, team competence, contingency planning, and adaptive capacity. A further resilience framework supports this through monitoring, anticipation, recognition, and learning, that are key to evolving after crises [27, 58, 72]. Based on these theoretical frameworks, Online Resource 3 presents the integrated theoretical foundation for the current systematic review.

In a nutshell, crises are characterized by ambiguity regarding their causes, effects, potential resolutions, and their significant impact on organizations, systems, or societies. However, the resilience framework, including the interrelated core components of monitoring, anticipation, recognition, and learning, provides an approach for building adaptive capacity in public sector organizations.

## Design and method

### Aim and research questions

This systematic review adopts an exploratory qualitative approach to synthesize qualitative and quantitative evidence on public organizations’ system-shock interventions, preparedness, and resilience interventions in the context of natural disasters driven by climate change. The systematic review is guided by the following research questions: (a) What types of organizational and community-based preparedness and resilience interventions exist for natural climate change-related crises? (b) How effective are these interventions in enhancing preparedness and resilience at the community and organizational levels? (c) What role does empowering education play in strengthening community and organizational resilience in response to natural climate change-related disasters and emergencies?

### Eligibility criteria

Studies were included in this review based on a set of predefined eligibility criteria. The target population comprised communities, organizations, and institutions that had implemented preparedness and resilience interventions in response to natural disasters associated with climate change. Eligible interventions were specifically designed to enhance preparedness and resilience in the context of climate-induced emergencies, such as floods, wildfires, or extreme weather events. As the review focused on qualitative synthesis, no comparison group was needed. Instead, emphasis was placed on reported outcomes, including the effectiveness of interventions, the role of empowering education, and indicators of both community and organizational resilience. The review considered empirical studies that employed either qualitative methods, such as interviews, focus groups, and case studies, or quantitative approaches, including correlational field studies. Only studies published in English were included, with no restrictions placed on the year of publication to ensure a comprehensive synthesis of available evidence. Studies were excluded if they were theoretical or conceptual in nature, appeared on Beall’s list of potential predatory journals and publishers, or were published in languages other than English.

### Search strategy

The review was conducted between March 2025 and June 2025. The systematic search considered the following databases: *Web of Science*, *Scopus*, *PsycINFO*, *ERIC*, and *Google Scholar* (to capture gray literature). The search terms included combinations and variations of the following keywords: ("climate change" OR "natural disaster" OR "climate crisis") AND ("preparedness" OR "resilience" OR "disaster management") AND ("community" OR "organization") AND ("education" OR "empowerment") AND ("intervention" OR "activities" OR "measures").

### Study selection process

Data extraction and synthesis followed a thematic analysis approach preregistered in the PROSPERO database. The selection process followed PRISMA guidelines and was conducted in two phases: first, a screening of titles and abstracts was performed, followed by a full-text review. The search results from all the databases were compiled into Excel, where duplicates were first removed by filtering and sorting functions, followed by manual review to ensure accuracy. The cleaned list of references was then imported into Rayyan [56] for blinded screening by the review team. Five independent reviewers assessed all studies for inclusion within the blind screening procedure. Any disagreements were resolved through discussion or, when necessary, by consulting a further reviewer of the authors involved.

### Data extraction and synthesis

Data were extracted via a standardized form that captured key information, including *authors, year of publication, country, target population, sample research design, main research questions, program/intervention, disaster/crisis and type, year of system shock, main results, preparation strategies, and resilience strategies*. The thematic synthesis approach was employed to identify recurring patterns, themes, and insights across the included studies. This method enables the integration of diverse data types, supporting the development of a fine-grained understanding of intervention effectiveness and the contribution of educational empowerment to resilience-building efforts. Online Resource 4 presents this form in the respective column head.

### Sampling and case selection strategy

A purposive sampling strategy was employed to ensure the inclusion of studies that directly addressed the research questions. This approach enabled the intentional selection of relevant and information-rich cases. In addition, a diverse case strategy was applied to capture a broad spectrum of interventions across various organizational and community contexts, geographic regions, and institutional settings.

### Risk of bias and credibility assessment

The methodological quality of the qualitative studies included was assessed using the Critical Appraisal Skills Program (CASP) checklist and formed part of the study selection process. Studies were excluded when they showed fundamental methodological shortcomings, such as unclear research aims, insufficient description of data collection or analysis, or a lack of coherence between data, interpretation, and conclusions. CASP appraisal was therefore applied as a threshold criterion rather than as a basis for differential weighting of included studies.

Further strategies to increase the credibility and trustworthiness of the review included triangulation with other data sources, the incorporation of multiple perspectives, independent analysis by different researchers, and cross-checking for rival explanations. Reflexivity was also maintained through regular team discussions and reflection on positionality. These strategies were particularly appropriate given the exploratory nature of the review and the focus on complex, context-dependent interventions. Together, these factors contributed to the rigor and depth of the synthesis.

### Assumptions about missing or unclear information

Where contextual information was incompletely reported, predefined assumptions were applied to ensure consistent synthesis. Studies in which the Year of Event was not specified, referred to ongoing or annual hazards, or coincided with data collection or training periods were coded as addressing recurrent hazard exposure rather than single events. Empirically similar studies from comparable contexts were retained as separate entries when publication and design differed. Several theoretically relevant moderators, including household composition, pre‑disaster social resources, and social support, could not be systematically coded due to inconsistent reporting and are therefore treated as unmeasured moderators.

### Stopping criteria

Following the guidance of Hennink and Kaiser [37], data saturation was considered to have been achieved when sufficient information was available to replicate the study, and no new insights emerged from additional data. At this point, the number of duplicates found increased.

### Data analysis and results report

**Fig. 1:**
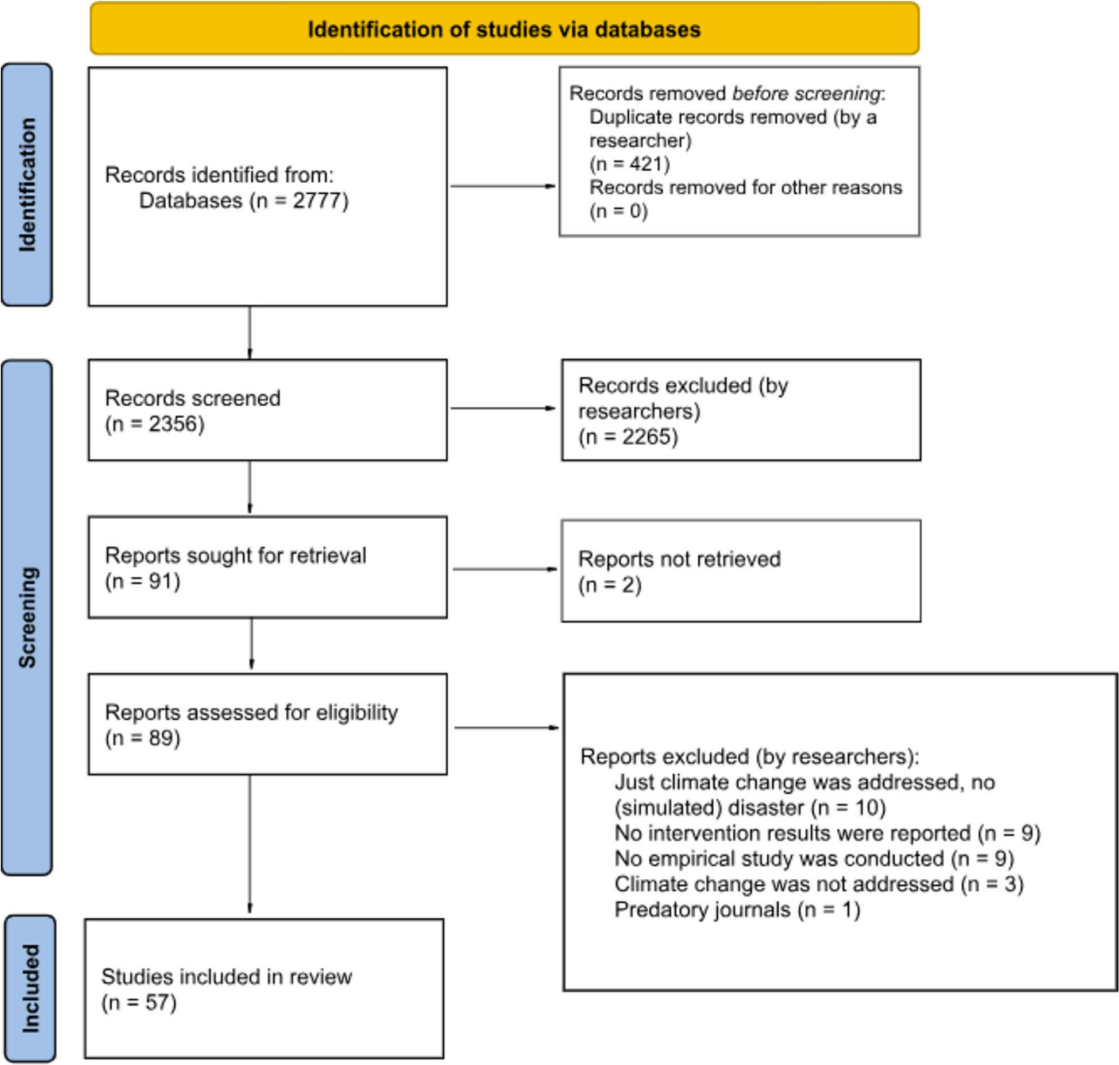
PRISMA flow diagram presenting the stages and processes from searching to including primary studies for synthesis [57]

The data analysis followed a systematic qualitative synthesis approach involving APA standards. The thematic synthesis was used to analyze heterogeneous data, enabling the identification of recurring patterns, themes, and conceptual insights across studies. Sample sizes per country are provided to highlight the regions worldwide where samples have been drawn. Based on sample characteristics, including sample type, sample size, study design (e.g., quantitative versus qualitative), and the use of control or comparison groups, a confidence rating was assigned to each study. Studies were ranked on a scale from 0 (excluded) to 4 (highest confidence). Higher ratings were given to studies with probabilistic sampling strategies or sufficiently large sample sizes to ensure robust statistical power, while lower ratings reflected limitations in design, sampling, or analytical rigor. The analysis focused on identifying the types of interventions implemented (before or after disasters or anticipated system shocks), their reported effectiveness, and the mechanisms through which empowering education strengthened resilience at both the community and organizational levels. We used the template provided by Jones [45] to report the results of this systematic review. Fig. 1 presents the PRISMA flow diagram.

## Results

This preregistered and theoretically founded qualitative systematic review mapped (a) which preparedness and resilience interventions exist in organizational and community settings (i.e., *types*), (b) if the authors concluded that these interventions were *effective* in enhancing resilience, and (c) the role of empowering education in strengthening adaptive capacity in response to climate-related disasters. Online Resource 3 presents the theoretical foundation. Online Resource 4 provides an overview of the country, sample, evidence from statistical analyses (yes/no), information about gender, age, socioeconomic status (yes/no), research design, and intervention for each of the 57 included studies. Online Resource 4 also presents the key findings and resilience strategies from each study. In addition, a confidence-scaled version of the results table is included on Page 2 of Online Resource 4.

The empirical studies identified in the systematic review can be meaningfully positioned within the crisis matrix proposed by Gundel [35] and extended through EU civil‑protection functions. The evidence spans conventional, unexpected, intractable, and fundamental crises, thereby empirically validating the multidimensional nature of the proposed framework. This qualitative synthesis is detailed next.

### Types and reported effectiveness of organizational and community-based preparedness and resilience interventions

The reviewed studies describe different types of preparedness and resilience interventions that cluster across the four crisis types of the theoretical framework (see Online Resource 3), reflecting varying levels of predictability and influencability. Interventions regarding Quadrant 1 “Conventional Crises” (i.e., predictable and influenceable) are primarily structured, education‑ and training‑based approaches aimed at strengthening preparedness under stable hazard conditions. These include curriculum‑integrated disaster risk reduction in schools [46*], theory‑based preparedness programs targeting behavioral change [41*], and school‑based risk education modules such as flood preparedness training [15*]. In addition, targeted training for professionals, such as climate‑health education programs, supports knowledge transfer and preparedness in institutional contexts [75*]. These interventions are typically standardized and focus on awareness, skills, and preventive practices.

Interventions regarding Quadrant 2 “Unexpected Crises” (i.e., less predictable but still influenceable, see Online Resource 3) emphasize adaptive preparedness, participatory learning, and decision‑making under uncertainty. Examples include serious games simulating climate‑related urban risks, allowing participants to experiment with alternative planning strategies [31*]. Further examples include participatory, place‑based climate education programs that link local experiences to broader environmental processes [16*]. Behaviorally informed approaches, such as automatic enrolment in preparedness schemes, also fall into this category, as they aim to increase uptake under uncertain conditions [13*]. These interventions are characterized by flexibility, experiential learning, and responsiveness to dynamic risk environments.

Studies regarding Quadrant 3 “Intractable Crises” (i.e., predictable but difficult to influence) describe structural, livelihood‑based, and system‑level interventions addressing long‑term vulnerabilities. These include adaptation strategies among smallholder farmers, such as irrigation, crop diversification, and soil conservation, shaped by access to resources and institutional conditions [9*]. Social enterprise approaches also illustrate efforts to address systemic climate challenges through sustainable agriculture, renewable energy, and partnership‑based models, while facing structural constraints such as policy instability and market pressures [5*]. These interventions highlight the limits of individual‑level preparedness and the importance of governance, infrastructure, and economic resources.

Interventions regarding Quadrant 4 “Fundamental Crises” (i.e., low predictability and low influencability) are described as community‑embedded, recovery‑oriented, and socially driven processes that emerge in response to highly disruptive events. Studies highlight the role of community participation, social networks, and informal coping strategies in disaster management and recovery [7*, 8*]. Additional approaches include large‑scale community initiatives integrating psychosocial support and collective engagement [24*], as well as communication‑based interventions such as storytelling and documentary formats that foster awareness and civic participation [44*]. These approaches emphasize social cohesion, collective action, and adaptive learning rather than predefined intervention structures.

Across the reviewed studies at the Confidence Level 4 (see Online Resource 4, p. 2), most interventions are reported as yielding statistically or descriptively improvements in knowledge, preparedness, participation, or resilience‑related outcomes, including educational, behavioral, and community‑based approaches [6*, 13*, 16*, 21*, 31*, 41*, 44*, 46*, 47*, 75*]. Partial or domain‑specific limitations were described in some cases, such as unchanged self‑efficacy [15*] or context‑dependent constraints related to socio‑economic conditions and observational designs [5*, 9*, 24*, 66*].

More specifically, interventions are described as contributing to changes in knowledge, awareness, and preparedness‑related behaviors, although outcomes vary by context and design. Educational and training interventions consistently report increases in knowledge, risk perception, and preparedness awareness among diverse groups, including professionals, students, and community members [15*, 41*, 75*]. Interactive and simulation‑based formats are described as supporting decision‑making capacities and understanding of complex trade‑offs in disaster risk contexts [31*].

Behavioral and structural interventions show that participation and uptake of preparedness measures are shaped by socio‑economic conditions, prior experience, and perceived risk, with mechanisms such as automatic enrolment influencing engagement differently across groups [13*]. Community‑based studies describe preparedness and recovery as strongly linked to social networks, participation, and local knowledge, while highlighting limitations in formal institutional support [7*, 8*]. At the same time, system‑level interventions emphasize the importance of institutional capacity, governance frameworks, and access to resources, with implementation challenges related to policy environments, market conditions, and infrastructure constraints [5*, 9*]. Moreover, the reviewed studies point to several challenges. These challenges fall into three main categories: (1) *institutional and regulatory gaps* (e.g., [4*, 5*, 7*], (2*) leadership and resource constraints* (e.g., [10*], and (3) *institutional resistance to change* (e.g., [14*].

### Role of empowering education in strengthening community and organizational resilience in response to natural climate change-related disasters and emergencies

Empowering education emerges as a central cross‑cutting element across intervention types. Studies describe educational approaches as facilitating knowledge development, awareness, and the capacity to engage with climate‑related risks, particularly when they are participatory, place‑based, and tailored to local contexts [16*, 46*]. Interactive formats, such as simulations, serious games, and interprofessional learning, are described as enabling experiential understanding and reflection on complex system dynamics [31*, 75*].

Education is also linked to communication and dissemination processes, including the use of media tools, storytelling, and public engagement formats, which support knowledge sharing and community dialogue [44*, 69*]. Several studies highlight that educational initiatives are embedded within broader social and organizational contexts and may be constrained by structural factors such as socio‑economic barriers, institutional resources, and community support [13*, 16*].

### Summary of reported gender, age, and socioeconomic status

Socio‑demographic characteristics were reported inconsistently across the studies summarized in Online Resource 4, Page 2, with age and gender most frequently documented in higher‑confidence quantitative and quasi‑experimental designs [13*, 31*, 41*, 46*, 66*, 75*]. Gender was shown to influence preparedness: women followed disaster news more closely [8*], and girls in Mexico’s Climate Change Education Program showed greater gains in knowledge and concern [16*]. Moreno and Shaw [53*] found shifts in gender roles after the 2010 Chile disaster.

Age or life stage defines target groups (e.g., children, youth, and adults; [66*, 74*], with higher age linked to lower participation in mitigation [8*]. Socioeconomic factors are predictors of preparedness [8*] and barriers to youth action despite increased behavioral intentions [16*].

Studies focusing on households, workers, farmers, or livelihood‑dependent populations were more likely to include socio‑economic indicators such as income, education, or occupation, demonstrating that socio‑economic inequalities shape preparedness, participation, and adaptation outcomes [8*, 9*, 19*, 47*]. In contrast, many education-, training-, and awareness-based interventions limited socio-demographic reporting to age and gender, despite targeting structurally diverse and often marginalized groups [15*, 16*, 25*, 69*].

Where socio‑economic status was explicitly examined, findings consistently revealed differential effects, including lower uptake, participation, or perceived benefits among lower‑income or less‑resourced groups [13*, 44*, 54*, 55*]. This synthesis points to a systematic gap in socio‑economic reporting, limiting cross‑study comparability and constraining conclusions regarding equity, vulnerability, and differential resilience outcomes across populations [24*, 30*, 73*].

### Risk of bias summary based on study characteristics

The reviewed evidence shows substantial methodological heterogeneity, indicating an elevated risk of bias across studies. Non‑experimental designs, including descriptive surveys and qualitative case studies, are particularly susceptible to selection, confounding, and interpretation biases, while reliance on self‑reported data increases the risk of recall and social desirability bias. Heterogeneity arises from variation in target populations, disaster types, intervention scope, and analytical rigor, ranging from acute disasters to chronic climate impacts and from small‑scale educational interventions to system‑level programs. Consequently, the overall certainty of the evidence informing strong policy recommendations is constrained by limitations in the primary studies. Online Resource 5 provides an overview of the studies excluded during the internal peer review conducted by Rayyan.

## Discussion

The systematic synthesis yielded that the described interventions can be meaningfully positioned within the crisis matrix (see Online Resource 3; [35, 52], reflecting differing levels of predictability and influencability. Regarding (a), interventions range from structured, education‑ and training‑based approaches in more predictable contexts to adaptive, community‑based and system‑level strategies in less predictable or harder‑to‑influence crises [6*, 8*, 13*, 66*].

In relation to (b), the findings suggest that reported preparedness and resilience outcomes are linked less to specific intervention types and more to their embedding within local conditions, governance arrangements, and social resources, consistent with the framework’s emphasis on anticipate, prepare, respond, and secure functions (e.g., [7*]. Interventions that remain detached from local contexts appear constrained in their contribution to these functions. With respect to (c), empowering education is described as a cross‑cutting mechanism operating across all quadrants, supporting learning, participation, and risk communication, particularly where linked to context‑sensitive and socially embedded practices [75*]. Thus, the studies portray resilience of communities and organisations as a context‑dependent and socially embedded process, shaped by the interaction between crisis characteristics, governance structures, and ongoing learning processes.

### Limitations of the evidence included in the review

Despite its breadth, the evidence base has methodological and structural limitations. Many studies rely on descriptive or quasi-experimental designs without control groups (see Online Resource 4). Small samples, such as nine university staff in New Zealand [14*] and 10 informants in South Africa [43*], offer rich insights but limited validity. Self-report bias is also a concern [7*]. High heterogeneity across contexts further limits applicability.

### Limitations of the review processes used

The review process, while mapping a diverse evidence base, lacks the key methodological steps needed to assess the certainty of findings. Study designs were only described (e.g., “qualitative case study”, see Online Resource 4). The wide variation in target groups and interventions did not allow for exploring whether differences in country, disaster type, or method explained outcome variation. The review functions primarily as evidence mapping. While promising strategies, such as community-led adaptation and tailored education, are highlighted, the lack of large-scale population-representative research or experimental rigor limits confidence in effectiveness.

### Implications for practice

Practice would benefit from context‑specific education that integrates knowledge, risk perception, and practical preparedness, while explicitly addressing gaps in self‑efficacy in responding to an unexpected crisis. Behaviorally informed approaches, such as automatic enrollment or simplified access to preparedness resources (if available), can increase uptake. Finally, strengthening community‑based and participatory approaches, including social networks, storytelling for sharing helpful strategies, and psychosocial support, is key to sustaining preparedness and collective resilience.

### Implications for policy

Policy should move beyond isolated measures toward coordinating enabling conditions at local and regional levels, including stable governance structures, financing mechanisms, and infrastructure that support sustained preparedness and adaptation. There is also a need to address structural barriers and inequalities, ensuring that preparedness resources and participation opportunities are accessible and responsive to diverse local conditions. In addition, policy frameworks should strengthen institutional arrangements that facilitate collaboration between public authorities, communities, and organizations, enabling context‑sensitive continuity between preparedness, response, and long‑term recovery.

### Implications for future research

The current evidence reveals significant gaps that future research needs to address. One priority is to understand the barriers to behavioral adoption, especially among vulnerable youth. It is not enough to measure knowledge; research might explore why concern often fails to translate into action, particularly when structural barriers erode hope. Longitudinal studies are needed to evaluate the long-term effectiveness of adaptive strategies, distinguishing between temporary relief and sustainable resilience across anticipatory, absorptive, and adaptive capacities.

Further investigation into governance and financing models that can overcome institutional inertia and policy volatility, particularly for social enterprises and community-based actors, is also needed. Finally, demographic factors, such as the presence of household members with special needs or gendered patterns in disaster information consumption, might be explored as levers for targeted preparedness campaigns.

## Conclusion

This systematic review indicates that preparedness and resilience interventions for climate‑related natural hazards are diverse and often embedded in local social, institutional, and environmental contexts. Across studies, resilience of communities and organizations is described as grounded in education, community participation, governance structures, and available resources, rather than in isolated interventions. Future longitudinal research might examine how these elements can be integrated in context‑sensitive ways to support sustained adaptation, action competencies, and equitable resilience outcomes.

## Supplementary Information

Supplementary Material 1: Online Resource 1: Preregistration protocol.

Supplementary Material 2: Online Resource 2: PRISMA checklist.

Supplementary Material 3: Online Resource 3: Theoretical framework for the systematic review.

Supplementary Material 4: Online Resource 4: Tables of results [3, 11, 12, 17, 18, 20, 22, 23, 29, 32–34, 39–42, 48–51, 60–65, 70, 71, 76].

Supplementary Material 5: Online Resource 5: Excluded studies and rationale for exclusion.

## Data Availability

The preregistration form and data extracted from the included studies used in the review are available at (https://osf.io/q84cm/?view_only=ebe8d3bf85bd4c8ba77c9959dd0138c4). The dataset supporting the conclusions of this article is available in the OSF repository, (https://osf.io/q84cm/?view_only=ebe8d3bf85bd4c8ba77c9959dd0138c4).
