# Supplementary material for: Natural climate-change-related crises: a systematic review of organizational and community preparedness and resilience
Source: BMC Public Health. 2026 Jun 18;26:1903. doi: 10.1186/s12889-026-27846-8 (PMC13277276; doi:10.1186/s12889-026-27846-8)
Supplement: Supplementary file 1 — Supplementary Material 1: Online Resource 1: Preregistration protocol. [file 12889_2026_27846_MOESM1_ESM.pdf]

Natural Climate-Change-Related Crises: Organizational and Community Preparedness and Resilience

Pending approval Updates

Metadata

Study Information

Research Aims

1. Background and Rationale:
Climate change has intensified the frequency and severity of natural disasters, posing significant challenges to communities and organizations. Preparedness and resilience interventions aim to mitigate the impact of these crises by fostering adaptive capacities at different levels. While numerous studies investigate these interventions, a systematic synthesis of qualitative evidence is needed to understand the types, effectiveness, and role of empowering education in such initiatives.

2. Objectives: This systematic review aims to:
- identify and categorize different types of organizational and community-based preparedness and resilience interventions for natural climate-change-related crises,
- assess the reported effectiveness of these interventions in enhancing preparedness and resilience,
- explore the role of empowering education in strengthening community and organizational resilience.

If helpful, please select the type of aim (non-exhaustive list):

Exploring

Research question(s)

What types of organizational and community-based preparedness and resilience interventions exist for natural climate-change-related crises?
How effective are these interventions in enhancing preparedness and resilience at the community and organizational levels?
What role does empowering education play in strengthening community and organizational resilience in response to natural climate-change-related disasters and emergencies?

Anticipated Duration

Start date: 03/2025
End date: 06/2025

Design Plan

Study design

Quantitative and qualitative studies (e.g., correlational field studies, interviews, focus groups, case studies).

Sampling and case selection strategy

A purposive sampling strategy will be employed to ensure the inclusion of diverse and relevant studies that provide insights into preparedness and resilience interventions. This approach allows for the selection of studies that specifically address the research questions, ensuring a rich and comprehensive synthesis of qualitative and quantitative findings.

For case selection, a diverse case strategy will be used, incorporating studies from various organizational and community contexts to capture a broad spectrum of interventions, effectiveness levels, and educational empowerment strategies. This approach ensures that different types of interventions, geographic regions, and institutional frameworks are considered, facilitating a more comprehensive understanding of resilience-building practices.

Data Collection

Data source(s) and data type(s)

A systematic search will be conducted in the following databases:
Web of Science
Scopus
PsycINFO
ERIC
Google Scholar (for grey literature)

Data collection methods

Search terms will include variations of:("climate change" OR "natural disaster" OR "climate crisis") AND ("preparedness" OR "resilience" OR "disaster management") AND ("community" OR "organization") AND ("education" OR "empowerment") AND ("intervention" OR "activities" OR "measu[illegible]

Help

Study Selection Process
Screening will be conducted in two phases: title/abstract screening followed by full-text screening.

Two independent reviewers will assess studies for inclusion. Disagreements will be resolved by discussion or a third reviewer.

Data Extraction and Synthesis
Data will be extracted using a standardized form including study characteristics, intervention details, effectiveness outcomes, and role of education.
A thematic synthesis approach will be used to identify patterns and themes across studies.

Data collection tools, instruments or plans

not applicable

No files selected

Stopping criteria

We follow Fusch & Ness (2005) and interpret saturation to be reached when there is enough information to replicate the study and the ability to obtain new information has been attained.

Analysis Plan

Data analysis approach

We use the systematic synthesis approach and follow the APA standards.

Data analysis process

3. Methodology

3.1 Eligibility Criteria

Population: Communities, organizations, and institutions implementing preparedness and resilience interventions.

Intervention: Preparedness and resilience interventions related to natural climate-change-induced disasters, emergencies, and challenges.

Comparison: Not applicable (qualitative synthesis).

Outcomes: Effectiveness, empowerment through education, community resilience, organizational preparedness.

Study Design: Quantitative and qualitative studies (e.g., correlational field studies, interviews, focus groups, case studies).

Language: English only.

Time Frame: No restrictions on publication year.

Exclusion Criteria: Theoretical contributions, publications within "Beall's List – of Potential Predatory Journals and Publishers", contributions published in other languages than English

3.2 Search Strategy
A systematic search will be conducted in the following databases:

Web of Science

Scopus

PsycINFO

ERIC

Google Scholar (for grey literature)

Search terms will include variations of:("climate change" OR "natural disaster" OR "climate crisis") AND ("preparedness" OR "resilience" OR "disaster management") AND ("community" OR "organization") AND ("education" OR "empowerment") AND ("qualitative study" OR "interview" OR "focus group" OR "case study").

3.3 Study Selection Process

Screening will be conducted in two phases: title/abstract screening followed by full-text screening.

Two independent reviewers will assess studies for inclusion. Disagreements will be resolved by discussion or a third reviewer.

3.4 Data Extraction and Synthesis

Data will be extracted using a standardized form including study characteristics, intervention details, effectiveness outcomes, and role of education.

A thematic synthesis approach will be used to identify patterns and themes across studies.

3.5 Sampling and Case Selection StrategyA purposive sampling strategy will be employed to ensure the inclusion of diverse and relevant studies that provide insights into preparedness and resilience interventions. This approach allows for the selection of studies that specifically address the research questions, ensuring a rich and comprehensive synthesis of qualitative and quantitative findings.

For case selection, a diverse case strategy will be used, incorporating studies from various organizational and community contexts to capture a broad

spectrum of interventions, effectiveness levels, and educational empowerment strategies. This approach ensures that different types of interventions, geographic regions, and institutional frameworks are considered, facilitating a more comprehensive understanding of resilience-building practices.

4. Risk of Bias Assessment

The Critical Appraisal Skills Programme (CASP) checklist for qualitative research will be used to assess methodological quality.

Credibility strategies

Triangulation with other data sources

Bringing in different perspectives

Have different researchers analyse the data

Cross-checks for rivalling explanations

Reflexitivity

Personal Responsibility

Please provide a short rationale for why you selected particular strategies and how they are appropriate given your study's aim(s) and approach, or specify your credibility strategies if not on the above list.

Rationale for Strategy Selection:
Purposive sampling is appropriate for this review because it allows for the intentional selection of studies that provide the most relevant and insightful information on preparedness and resilience interventions. Given the focus on understanding intervention effectiveness and empowerment through education, ensuring a diverse representation of cases enables a more comprehensive analysis of contextual influences. The diverse case strategy is particularly useful in capturing variations across different community and organizational settings, allowing for a richer synthesis of best practices and challenges in implementing resilience-building initiatives.

Miscellaneous

Reflection on your positionality (optional)

We conduct focus groups to discuss different perspectives.

Copyright © 2011-2025 Center for Open Science | Terms of Use | Privacy Policy | Status | API
TOP Guidelines | Reproducibility Project: Psychology | Reproducibility Project: Cancer Biology
