# Supplementary material for: Natural climate-change-related crises: a systematic review of organizational and community preparedness and resilience
Source: BMC Public Health. 2026 Jun 18;26:1903. doi: 10.1186/s12889-026-27846-8 (PMC13277276; doi:10.1186/s12889-026-27846-8)
Supplement: Supplementary file 3 — Supplementary Material 3: Online Resource 3: Theoretical framework for the systematic review. [file 12889_2026_27846_MOESM3_ESM.docx]

# **Natural Climate-Change-Related Crises: Organizational and Community Preparedness and Resilience.**

# **A Systematic Review**

**Online Resource 3**

*Theoretical Framework for the Current Study*

| 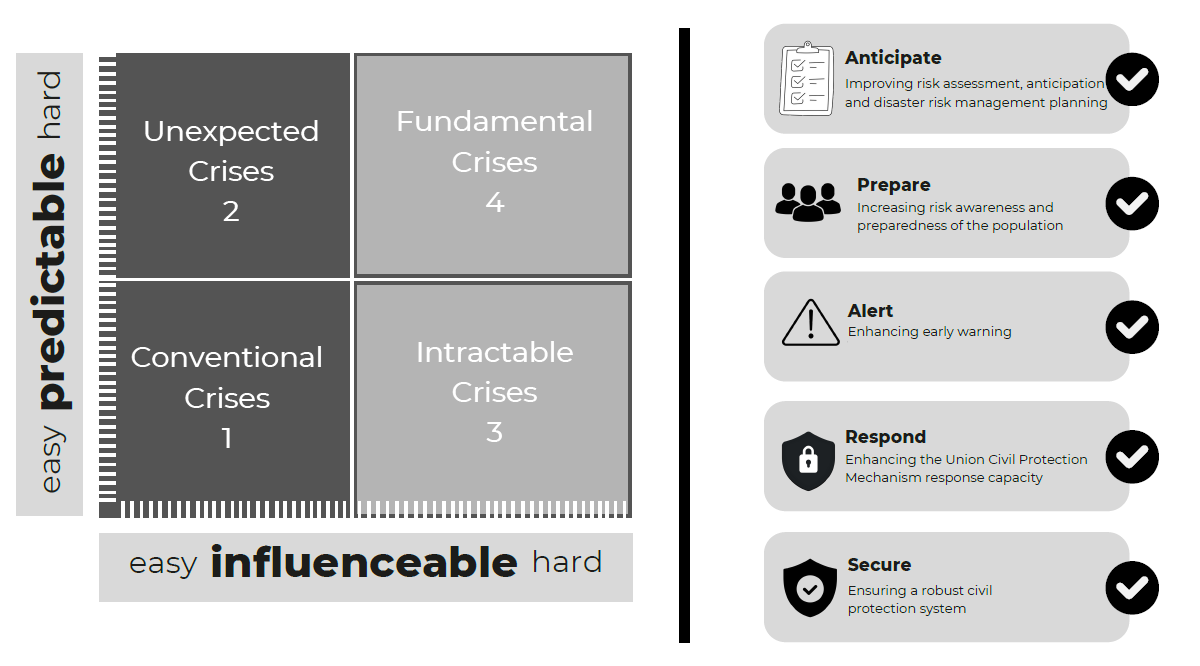 |
| --- |

*Note.* The framework integrates different approaches (European Commission, 2025; Mitroff et al., 1987) into the crisis matrix proposed by Gundel (2005).
